# 4-Octyl Itaconate Prevents Free Fatty Acid-Induced Lipid Metabolism Disorder through Activating Nrf2-AMPK Signaling Pathway in Hepatocytes

**DOI:** 10.1155/2022/5180242

**Published:** 2022-02-18

**Authors:** Xu Chu, Longlong Li, Weiyuan Yan, Haitian Ma

**Affiliations:** ^1^Key Laboratory of Animal Physiology and Biochemistry, College of Veterinary Medicine, Nanjing Agricultural University, Nanjing 210095, China; ^2^MOE Joint International Research Laboratory of Animal Health and Food Safety, College of Veterinary Medicine, Nanjing Agricultural University, Nanjing 210095, China

## Abstract

Nonalcoholic fatty liver disease (NAFLD), characterized with oxidative stress and hepatic steatosis, is a serious threat to human health. As a specific activator of nuclear factor E2-related factor 2 (Nrf2), the 4-octyl itaconate (4-OI) has the beneficial effects in antioxidant and anti-inflammation; however, whether 4-OI can alleviate hepatic steatosis and its mechanism is still unknown. The present study was aimed at investigating the protective effects of 4-OI on free fat acid- (FFA-) induced lipid metabolism disorder and its potential molecular mechanism in hepatocytes. The results showed that 4-OI treatment markedly alleviated FFA-induced oxidative stress and excessive lipid accumulation in hepatocytes. Mechanistically, 4-OI significantly suppressed the overproduction of reactive oxygen species (ROS) through activation of Nrf2; the downregulation of ROS level induced a downregulation of AMP-dependent protein kinase (AMPK) phosphorylation level which finally ameliorated excessive lipid accumulation in FFA-stimulated hepatocytes. In general, our data demonstrated that 4-OI relieves the oxidative stress and lipid metabolism disorder in FFA-stimulated hepatocytes; and these beneficial effects were achieved by activating the Nrf2-AMPK signaling pathway. These data not only expand the new biological function of 4-OI but also provide a theoretical basis for 4-OI to protect against lipid metabolism disorders and related diseases, such as NAFLD.

## 1. Introduction

Nonalcoholic fatty liver disease (NAFLD) is mainly caused by intemperate diet and irregular lifestyle, which seriously threatens human health [1]. In recent years, the prevalence of NAFLD has generally increased in the world [2] and has become a precursor of liver fibrosis, liver cirrhosis, and even liver cancer [3, 4]. Previous studies found that NAFLD not only associates with the increase of liver-related morbidity but also affects the occurrence of many metabolic diseases [5, 6]. The pathogenesis of NAFLD is summarized as “multihit hypothesis,” including the excessive liver fat deposition that causes oxidative stress and inflammatory response, which finally exacerbates hepatic steatosis [7]. Currently, there is no effective strategy for the prevention of NAFLD; most bioactive substance or metabolic intermediates exhibit a multitudinous potential effects; employing it to reduce fat accumulation, oxidative stress, or inflammatory response may be an effective strategy for alleviating NAFLD and other metabolic diseases.

Oxidative stress is caused by the imbalance of the redox system in the pathological state of the body [8]. Nuclear factor E2-related factor 2 (Nrf2) has been proved to mediate the antioxidant factor expression, which is the main pathway in regulating oxidative stress and electrophilic stress [9]. Under stress conditions, Nrf2 is combined with an antioxidant response element to initiate the transcription of related genes and improve the antioxidant capacity, which then reduces the production of reactive oxygen species (ROS) in the body [10, 11]. It is reported that oxidative stress leads to high levels of ROS production; and the activation of AMP-dependent protein kinase (AMPK) is affected by ROS level [12–14]. As an important energy sensor and regulator, AMPK plays an important role in regulating lipid deposition [15, 16]; thus, activated AMPK maybe as a potential target in preventing NAFLD and related metabolic diseases.

Itaconic acid is generated from the decarboxylation of tricarboxylic acid cycle (TCA) intermediate cis-aconitic acid by the catalyzing of immune response gene 1 in macrophages [17]; and it has protective effects on oxidative stress and inflammatory response [18, 19]. Previous study have shown that itaconic acid can directly alkylate cysteine residue on Kelch-like ECH-associated protein 1 (Keap1), thereby activating Nrf2 to exert antioxidant and anti-inflammatory effects [20]. At present, research on itaconic acid has mainly focused on its immunomodulation, and it has been found that it has a notable anti-inflammatory effect on activated macrophages [21, 22]. As an unsaturated dicarboxylic acid, itaconic acid is difficult to penetrate cell membrane and is not suitable for a mechanism study. Thus, 4-octyl itaconate (4-OI), a cell-permeable itaconic acid derivative [23], is widely used in the corresponding molecular studies. It is reported that 4-OI exerts metabolic regulatory by affecting the activity of key enzymes in the TCA cycle [24]; and it can reduce the supply of energy substances by interfering with the glycolysis pathways, reducing energy consumption and fat deposition [25]. Moreover, 4-OI can also exert antioxidant effects by inhibiting ROS production in hepatocytes [25]. However, whether 4-OI can alleviate the oxidative stress and lipid metabolism disorder in hepatocytes is still unknown.

Therefore, the present study was aimed at verifying the protective effects and molecular mechanisms of 4-OI against free fat acid- (FFA-) stimulated lipid metabolism disorder and oxidative stress in the BRL-3A and LO2 cells. We found that 4-OI significantly blocked the overproduction of ROS through activating Nrf2, leading to the activation of AMPK signaling, which then alleviated excessive lipid accumulation in FFA-stimulated hepatocytes. These findings not only reveal novel biological function of 4-OI but also provide a theoretical basis for its use as a nutritional supplement to protect against metabolism disorders and related diseases such as NAFLD.

## 2. Materials and Methods

### 2.1. Chemicals

4-Octyl itaconate (4-OI), dimethyl sulfoxide (DMSO), methyl thiazolyl tetrazolium (MTT), penicillin-streptomycin, and trypsin were purchased from Sigma (St. Louis, MO, USA). RPMI 1640 was purchased from Gibco (Grand Island, NY, USA). The TRIzol reagent kit was purchased from Invitrogen (Carlsbad, CA, USA). Ex Taq DNA polymerase was purchased from TaKaRa Bio Inc. (Shiga, Japan), and SYBR Green PCR Master Mix was purchased from Roche (Basel, Switzerland). The assay kits of superoxide dismutase (SOD, #mbs237a), peroxidase (POD, #mbca502), catalase (CAT, #mbe12189), total antioxidant capacity (T-AOC, #a015a), malondialdehyde (MDA, #a00312), and triglyceride (TG) were obtained from Nanjing Jiancheng Institute of Biotechnology (Nanjing, China). BCA protein assay kit was purchased from Beyotime Institute of Biotechnology (Shanghai, China). Compound C, NAC, and ML385 were purchased from Beyotime Biotechnology Institute (Shanghai, China). The rabbit anti-Nrf2 (A0647), Keap1 (A17062), AMPK (A1009), p-AMPK (AP0637), ACC1 (AG16452), p-ACC1 (A1346), and SREBP-1 (AB133125) antibodies were purchased from ABclonal Technology (Wuhan, China. Goat anti-rabbit IgG (H+L) (GB21303) secondary antibody was purchased from Wuhan Servicebio Technology Co., Ltd. (Wuhan, China); and goat anti-rabbit IgG (H+L) FITC (BL033A) antibody was obtained from Biosharp (Hefei, China).

### 2.2. Cell Culture and Treatment

Human liver LO2 cells (Hongsheng Biological Technology Co., Ltd., Nanjing, China) and rat liver BRL-3A cells (ATCC, Manassas, VA) were, respectively, cultured in RPMI 1640 and DMEM with 10% fetal bovine serum, 100 U/ml penicillin, and 100 *μ*g/ml streptomycin; and the cells were maintained in a humidified atmosphere of 5% CO_2_ air at 37°C. The LO2 cells and BRL-3A cells were pretreated with 4-OI for 4 h and then treated with 0.75 mM FFA (oleic acid: palmitic acid = 2 : 1) for another 24 h.

### 2.3. Cell Viability Assay

The LO2 cells and BRL-3A cells were seeded in 96-well plates (5 × 10^4^ cells/well) and then treated with 0, 12.5, 25, 50, or 100 *μ*M 4-OI for 4 h. Then, 10 *μ*l MTT (5 mg/ml in PBS) was added to each well and incubated at 37°C for 4 h; after that, the culture supernatant was removed, and 150 *μ*l DMSO was added to dissolve crystals. The absorbance was measured at 490 nm using a model 550 Microplate Reader (Bio-Rad, California, USA).

### 2.4. TG Content Analysis and Lipid Droplets Accumulation

The LO2 cells and BRL-3A cells were seeded into 6-well plates (1 × 10^6^ cells/well). BRL-3A cells were pretreated with or without 25, 50, and 100 *μ*M 4-OI for 4 h, and the LO2 cells were pretreated with or without 50 *μ*M 4-OI for 4 h; then, all cells were cultured with or without 0.75 mM FFA for another 24 h. The cells were collected, and TG contents were measured using the commercial kit, and the data were normalized to the protein concentration as determined by BCA protein assay kit following the manufacturers.

For the analysis of lipid droplet accumulation, Nile red staining is used in this study. Briefly, the cells were pretreated with or without 50 *μ*M 4-OI for 4 h and then cultured with or without 0.75 mM FFA for another 24 h. After that, the cells were stained with Nile Red staining solution at 37°C for 10 min and washed with PBS, and then, the cells were photographed using a Zeiss 710 Laser Scanning confocal microscope (Zeiss, Germany).

### 2.5. Antioxidant Capacity Analysis

The LO2 and BRL-3A cells were grown in 6-well plates (1 × 10^6^ cells/well). The BRL-3A cells were pretreated with or without 25, 50, and 100 *μ*M 4-OI for 4 h, and the LO2 cells were pretreated with or without 50 *μ*M 4-OI for 4 h; then, all cells were cultured with or without 0.75 mM FFA for another 24 h. The cells were washed and collected, disrupted ultrasonically in ice, and centrifuged at 2500 rpm for 10 min at 4°C. The activity of T-AOC, SOD, CAT, POD, and the content of MDA was determined following the commercial kit manufacturers, and the data were normalized with protein content that was determined using a BCA assay kit according to the manufacturers.

### 2.6. Reactive Oxygen Species (ROS) and Mitochondrial ROS (mROS) Analysis

Intracellular generation of ROS and mROS was detected with the probes of 2′,7′-dichlorodihydrofluorescein diacetate (DCFH-DA) and MitoSOX Red (Thermo, Waltham, MA, USA). Briefly, the LO2 cells and BRL-3A cells were pretreated with or without 50 *μ*M 4-OI for 4 h and then cultured with or without 0.75 mM FFA for another 24 h. Subsequently, the culture supernatant was removed, and the cells were treated with 10 *μ*M DCFH-DA probe or 5 *μ*M MitoSOX Red probe for 30 min in the dark. After that, the cells were washed with PBS and photographed using a Zeiss 710 Laser Scanning confocal microscope (Zeiss, Germany).

### 2.7. Measurement of Mitochondrial Membrane Potential

The mitochondrial membrane potential (MMP) was measured using JC-1 probe (Beyotime Institute of Biotechnology, Shanghai, China). In brief, the cells were seeded into 12-well plates (1 × 10^4^ cells/well) and preincubated with or without Nrf2 inhibitor 10 *μ*M ML385 for 12 h and then treated with or without 50 *μ*M 4-OI for 4 h; after that, the cells were treated with 0.75 mM FFA for another 24 h. After the experiment, the cells were incubated with JC-1 probe for 30 min at 37°C in the dark and then washed with JC-1 washing buffer; the samples were imaged using a Zeiss 710 Laser Scanning confocal microscope (Zeiss, Germany).

### 2.8. Real-Time Quantitative PCR Analysis

The total RNA was extracted and determined using the TRIZOL reagent kit as previously described [26]. Briefly, total RNA was reverse transcribed into cDNA using the Superscript II kit (Promega, USA) according to the manufacturers. The mRNA level was quantified by real-time quantitative PCR using a SYBR green PCR kit (Roche, Switzerland). All results were analyzed in duplicate using the IQ5 Sequence Detection System (Bio-Rad, California, USA). The 2^-△△CT^ method was used to calculate the fold change in the mRNA levels. The forward and reverse primers for sterol-regulatory element binding proteins-1c (*SREBP-1c*), fatty acid synthase (*FAS*), carnitine palmitoyl transferase-1 (*CPT-1*), peroxidase proliferation-activated receptor alpha (*PPARα*), heme oxygenase-1 (*HO-1*), NAD(P)H: quinone oxidoreductase 1 (*NQO1*), and glyceraldehyde-3-phosphate dehydrogenase (*GAPDH*) are displayed in Table 1. The *GAPDH* was used as the standard.

### 2.9. Western Blot Analysis

Total protein from the was extracted using NP40 lysis buffer and quantified by BCA assay following introduction of the manufacturers and then separated using 10% SDS-PAGE gels. Then, the extracted protein were transferred onto PVDF members and then blocked with 5% nonfat milk for 2 h. The membranes were incubated overnight at 4°C with rabbit antibodies against the Nrf2, Keap1, AMPK, p-AMPK, ACC1, p-ACC1, and SREBP-1. After washing, the membranes were incubated with secondary antibody for another 2 h at room temperature. The ECL enhanced chemiluminescence was used as a detecting agent, and the protein abundance was quantified by the ImageJ software.

### 2.10. Statistical Analysis

The GraphPad Prism 5 software was performed to analysis the data, and the one-way ANOVA and Student-Newman-Keuls test were used for comparing the significant differences among different treatments. All of the results were obtained from at least three independent experiments.

## 3. Results

### 3.1. 4-OI Alleviates FFA-Induced Lipid Accumulation in Hepatocytes

**Figure 1:**
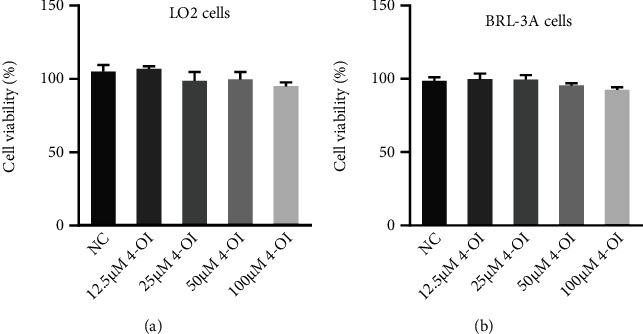
Effects of 4-OI on cell viability in the LO2 and BRL-3A cells. The LO2 cells and BRL-3A cells were cultured with 0, 12.5, 25, 50, or 100 *μ*M 4-octyl itaconate (4-OI) for 4 h. Viability of the LO2 cells (a) or BRL-3A cells (b) under treated with different doses of 4-OI. Data are presented as the means ± SEM.

As shown in Figure 1, no significant differences were observed on the cell viability in the LO2 cells and BRL-3A cells treated with 12.5-100 *μ*M 4-OI, which implied that the doses of 4-OI used in this experiment will not cause obvious cell toxicity.

**Figure 2:**
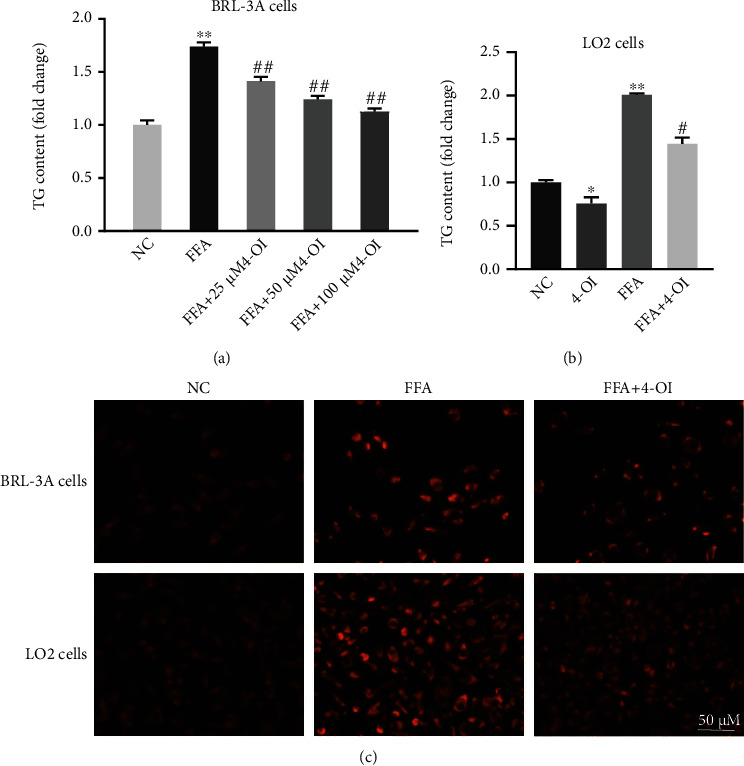
4-OI alleviates FFA-induced lipid accumulation in hepatocytes. The BRL-3A cells were pretreated with or without 25, 50, and 100 *μ*M 4-octyl itaconate (4-OI) for 4 h, and the LO2 cells were pretreated with or without 50 *μ*M 4-OI for 4 h; then, all cells were treated with or without 0.75 mM free fat acid (FFA) for another 24 h. (a) Triglyceride (TG) content in the BRL-3A cells. (b) TG content in the LO2 cells. (c) Representative pictures of Nile red staining in the BRL-3A cells (A–C) and LO2 cells (D–F). Data are presented as the means ± SEM. ^∗^*P* < 0.05 and ^∗∗^*P* < 0.01, compared to the NC group. ^#^*P* < 0.05 and ^##^*P* < 0.01, compared to the FFA group.

We found that the intracellular TG content was significantly increased in FFA-induced cells, while 4-OI treatment effectively reduced the TG content than the FFA-stimulated group in the LO2 and BRL-3A cells (Figures 2(a) and 2(b)). To further certify these results, the Nile Red staining was used to evaluate the lipid droplets accumulation, and the results showed that lipid droplet accumulation was obviously increased in the FFA-induced LO2 cells and BRL-3A cells when compared to the control group, while 4-OI treatment effectively attenuated the lipid droplet accumulation in hepatocytes (Figure 2(c)). These results illustrated that 4-OI alleviates the lipid accumulation in the FFA-stimulated hepatocytes.

### 3.2. 4-OI Relieves Lipid Accumulation through Activated AMPK Pathway in Hepatocytes

**Figure 3:**
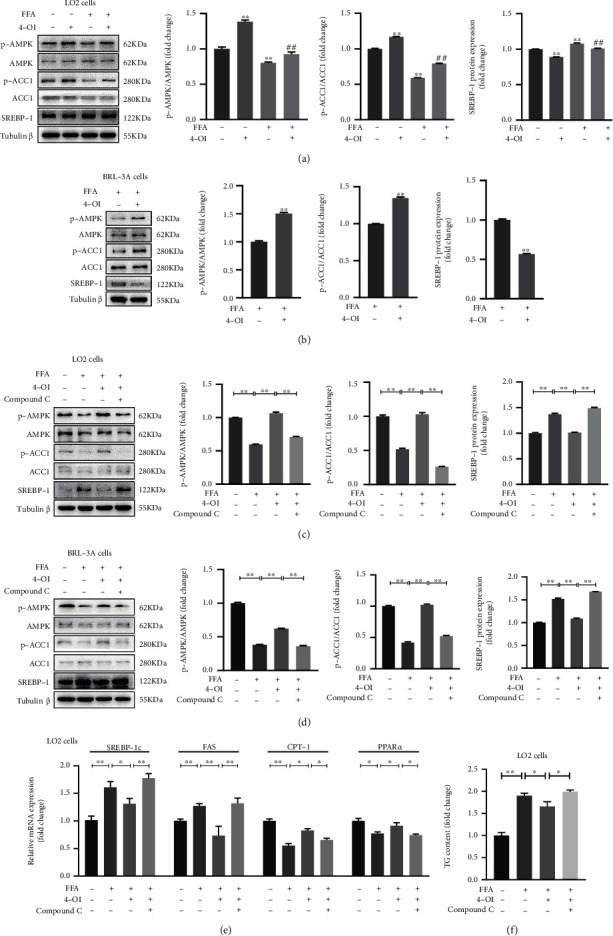
4-OI relieves lipid accumulation through activation of AMPK signaling pathway in hepatocytes. The LO2 cells and BRL-3A cells were pretreated with/without AMP-dependent protein kinase (AMPK) inhibitor compound C (10 *μ*M) for 1 h, then cultured with or without 50 *μ*M 4-octyl itaconate (4-OI) for 4 h, and then added with/without 0.75 mM FFA for another 24 h. (a) Immunoblotting and statistical analysis of p-AMPK, AMPK, p-ACC1, acetyl-CoA carboxylase 1 (ACC1), and sterol-regulatory element binding proteins-1 (SREBP-1) in the LO2 cells. Data are presented as the means ± SEM. ^∗∗^*P* < 0.01, compared to the NC group. ^##^*P* < 0.01, compared to the FFA group. (b) Immunoblotting and statistical analysis of p-AMPK, AMPK, p-ACC1, ACC1, and SREBP-1 in the BRL-3A cells. Data are presented as the means ± SEM. ^∗∗^*P* < 0.01, compared to the FFA group. (c) Immunoblotting and statistical analysis of p-AMPK, AMPK, p-ACC1, ACC1, and SREBP-1 in the LO2 cells pretreated with AMPK inhibitor compound C. Data are presented as the means ± SEM. ^∗∗^*P* < 0.01, comparison between the indicated groups. (d) Immunoblotting and statistical analysis of p-AMPK, AMPK, p-ACC1, ACC1 and SREBP-1 in the BRL-3A cells pretreated with AMPK inhibitor compound C. Data are presented as the means ± SEM. ^∗∗^*P* < 0.01, comparison between the indicated groups. (e) The mRNA levels of *SREBP-1c*, *fatty acid synthase (FAS)*, *carnitine palmitoyl transferase-1 (CPT-1)*, and *peroxidase proliferation-activated receptor alpha (PPARα)* in the LO2 cells pretreated with AMPK inhibitor compound C. Data are presented as the means ± SEM. ^∗^*P* < 0.05 and ^∗∗^*P* < 0.01, comparison between the indicated groups. (f) TG content in the LO2 cells. Data are presented as the means ± SEM. ^∗^*P* < 0.05 and ^∗∗^*P* < 0.01, comparison between the indicated groups.

It is reported that AMPK plays a crucial role in lipid metabolism [27]. In this study, we found that 4-OI treatment significantly increased the AMPK and ACC1 phosphorylation protein level; but the SREBP-1 protein level was significantly decreased in the FFA-stimulated LO2 cells pretreated with 4-OI (Figure 3(a)). Meanwhile, 4-OI treatment also enhanced the phosphorylation protein level of AMPK and ACC1 and reduced SREBP-1 protein level in the FFA-stimulated BRL-3A cells (Figure 3(b)).

To further verify the actions of 4-OI regulating lipid metabolism whether associated with the activating of AMPK, the hepatocytes were preincubated with 10 *μ*M AMPK inhibitor compound C to block the phosphorylation of AMPK. The results showed that 4-OI induced the upregulation of phosphorylation of AMPK and ACC1 protein level and the downregulation of SREBP-1 protein level completely reversed in the FFA-stimulated LO2 cells and BRL-3A cells pretreated with compound C (Figures 3(c) and 3(d)). Meanwhile, the RT-qPCR assay also showed that 4-OI treatment significantly decreased the upregulation of *SREBP-1c* and *FAS* mRNA level and the downregulation of *CPT-1* and *PPARα* mRNA levels in the FFA-stimulated LO2 cells, and these actions of 4-OI were also evidently reversed in the FFA-stimulated LO2 cells pretreated with compound C (Figure 3(e)). Importantly, we found that the deceasing effect of TG content induced by of 4-OI treatment was disappeared in the FFA-stimulated LO2 cells pretreated with compound C (Figure 3(f)). These data implied that 4-OI relieves lipid accumulation via activating AMPK signaling pathway in hepatocytes.

### 3.3. 4-OI Activates AMPK Pathway by Preventing the Overproduction of ROS in Hepatocytes

**Figure 4:**
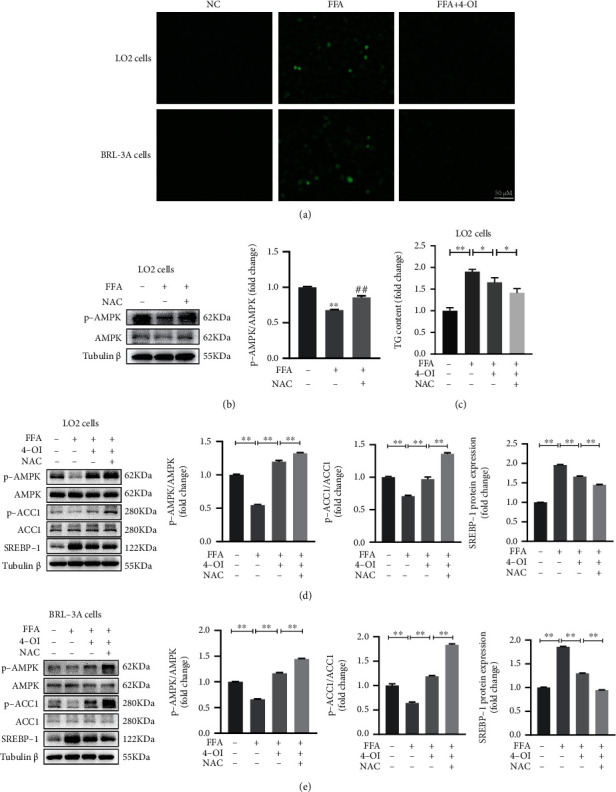
4-OI activates AMPK signaling pathway by reducing ROS production. The LO2 cells and BRL-3A cells were pretreated with/without reactive oxygen species (ROS) scavenger NAC (5 *μ*M) for 1 h, then cultured with or without 50 *μ*M 4-octyl itaconate (4-OI) for 4 h, and then added with/without 0.75 mM free fat acid (FFA) for another 24 h. (a) Representative pictures of ROS levels in the LO2 cells (A–C) and BRL-3A cells (D–F). (b) Immunoblotting and statistical analysis of p-AMP-dependent protein kinase (AMPK) and AMPK in the LO2 cells. Data are presented as the means ± SEM. ^∗∗^*P* < 0.01, compared to the NC group. ^##^*P* < 0.01, compared to the FFA group. (c) TG content in the LO2 cells. (d) Immunoblotting and statistical analysis of p-AMPK, AMPK, p-ACC1 (acetyl-CoA carboxylase 1), ACC1, and sterol-regulatory element binding proteins-1 (SREBP-1) in the LO2 cells pretreated with ROS scavenger NAC. (e) Immunoblotting and statistical analysis of p-AMPK, AMPK, p-ACC1, ACC1, and SREBP-1 in the BRL-3A cells pretreated with ROS scavenger NAC. (c–e) Data are presented as the means ± SEM. ^∗^*P* < 0.05 and ^∗∗^*P* < 0.01, comparison between the indicated groups.

The increase of intracellular ROS production was significantly blocked in the FFA-stimulated hepatocytes treated with 4-OI (Figure 4(a)). A series of studies have shown that the AMPK activation is related to ROS levels [12]. To verify the activation of AMPK induced by 4-OI whether it is related to the inhibition of ROS overproduction in the FFA-stimulated hepatocytes, we next detected the AMPK phosphorylation level in hepatocytes pretreated with 5 *μ*M ROS scavenger NAC. The results showed that NAC alone treatment obviously increased the AMPK phosphorylation protein level and decreased the TG content in the FFA-stimulated LO2 cells (Figures 4(b) and 4(c)). However, NAC and 4-OI treatments synergistically significantly decreased the TG content (Figure 4(c)) and SREBP-1 protein level (Figures 4(d) and 4(e)) and significantly enhanced the phosphorylation of AMPK and ACC1 protein level in the FFA-induced LO2 cells or BRL-3A cells (Figures 4(d) and 4(e)). Taken together, these results indicated that 4-OI can activate AMPK signaling pathway by reducing intracellular ROS overproduction in hepatocytes.

### 3.4. 4-OI Relieves FFA-Induced Oxidative Stress by Activated Nrf2 in Hepatocytes

**Figure 5:**
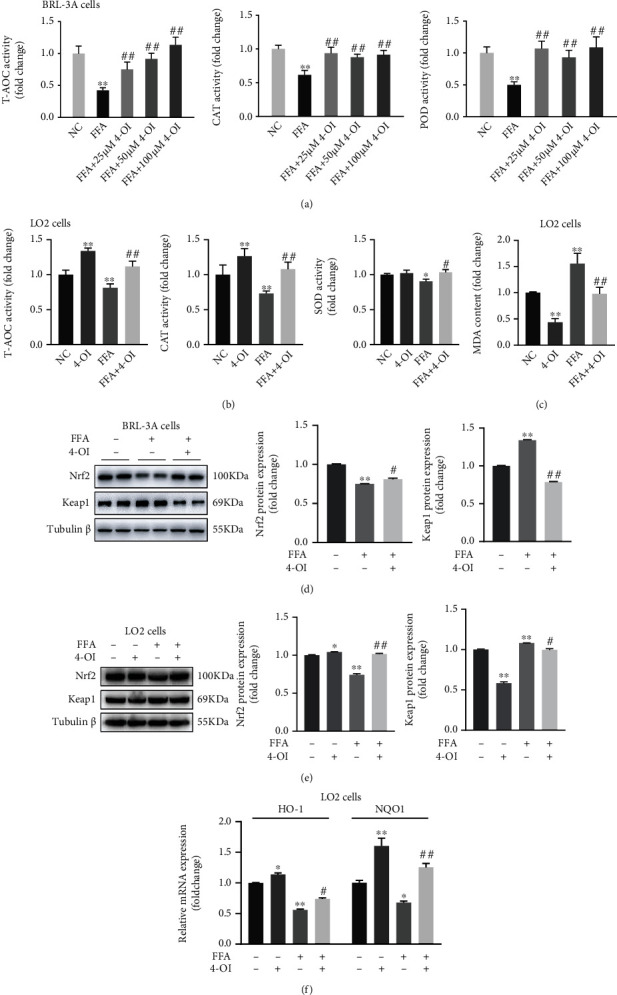
4-OI relieves FFA-induced oxidative stress through Nrf2 signaling pathway. The BRL-3A cells were pretreated with or without 25, 50, and 100 *μ*M 4-octyl itaconate (4-OI) for 4 h, and the LO2 cells were pretreated with or without 50 *μ*M 4-OI for 4 h; then, all cells were treated with or without 0.75 mM free fat acid (FFA) for another 24 h. (a) The activities of total antioxidant capacity (T-AOC), catalase (CAT), and peroxidase (POD) in the BRL-3A cells. (b) The activities of T-AOC, CAT, and superoxide dismutase (SOD) in the LO2 cells. (c) Malondialdehyde (MDA) content of the LO2 cells. (d) Immunoblotting and statistical analysis of nuclear factor E2-related factor 2 (Nrf2) and Kelch-like ECH-associated protein 1 (Keap1) in the BRL-3A cells. (e) Immunoblotting and statistical analysis of Nrf2 and Keap1 in the LO2 cells. (f) *Heme oxygenase 1 (HO-1)* and *NAD(P)H: quinone oxidoreductase 1 (NQO1)* mRNA levels in the LO2 cells. Data are presented as the means ± SEM. ^∗^*P* < 0.05 and ^∗∗^*P* < 0.01, compared to the respective control group. ^#^*P* < 0.05 and ^##^*P* < 0.01, compared to the FFA group.

The oxidation and antioxidant systems of the body are usually in dynamic balance and maintain the normal life activities [8]; once this dynamic equilibrium is broken, it is easy to cause oxidative stress. As shown in Figures 5(a) and 5(b), FFA alone treatment caused a significant decrease in the activities of T-AOC, CAT, and POD, which was significantly reversed in the LO2 cells and BRL-3A cells treated with 4-OI. In addition, the MDA content significantly increased after FFA treatment, and the addition of 4-OI treatment significantly reduced MDA content in the presence or absence of FFA in the LO2 cells (Figure 5(c)).

It well known that the Nrf2 cascade is a classic antioxidant pathway. In order to further explore the mechanisms of 4-OI alleviating the accumulation of ROS, we subsequently analyzed the effect of 4-OI on the Nrf2 signaling pathway. Western blot analysis showed that 4-OI treatment significantly alleviated the FFA-induced downregulation of Nrf2 protein level and upregulation of Keap1 protein level in the LO2 cells and BRL-3A cells (Figures 5(d) and 5(e)). Consistently, 4-OI treatment significantly increased the mRNA levels of *HO-1* and *NQO1* in the presence or absence of FFA in the LO2 cells (Figure 5(f)). Therefore, these results indicated that 4-OI can protect against the FFA-induced oxidative stress through activation of Nrf2 signaling pathway.

### 3.5. 4-OI Activates AMPK Pathway via Nrf2-Mediated Inhibition on mROS Production

**Figure 6:**
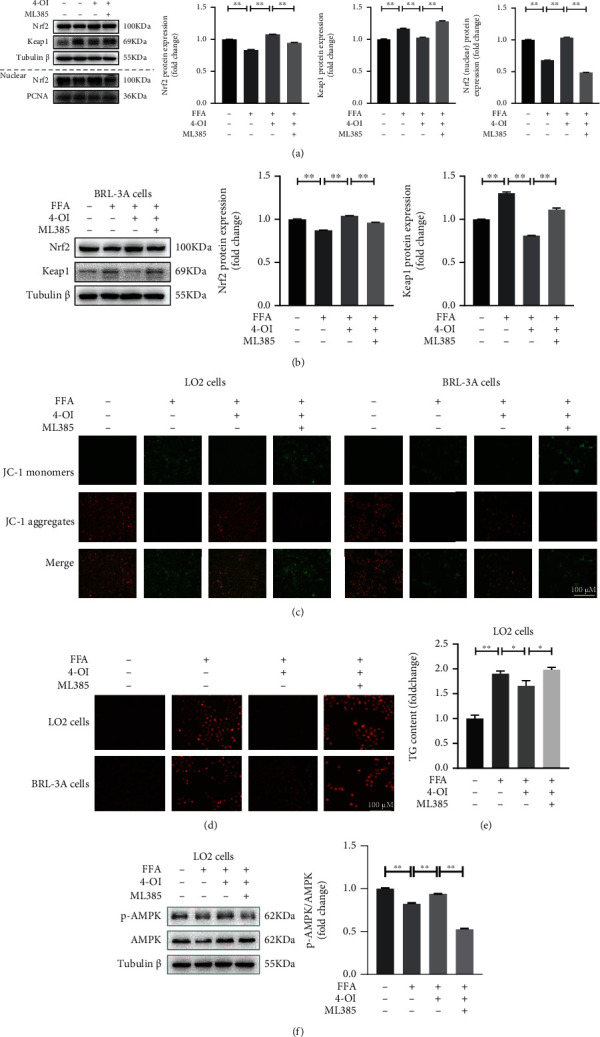
4-OI indirectly activates AMPK via activating the Nrf2 signaling pathway. The LO2 cells and BRL-3A cells were preincubated with/without Nrf2 inhibitor ML385 (10 *μ*M) for 12 h and then cultured with or without 50 *μ*M 4-octyl itaconate (4-OI) for 4 h, and then added with/without 0.75 mM free fat acid (FFA) for another 24 h. (a) Immunoblotting and statistical analysis of nuclear factor E2-related factor 2 (Nrf2), Kelch-like ECH-associated protein 1 (Keap1), and nuclear Nrf2 in the LO2 cells pretreated with Nrf2 inhibitor ML385. (b) Immunoblotting and statistical analysis of Nrf2 and Keap1 in the BRL-3A cells pretreated with Nrf2 inhibitor ML385. (c) Representative pictures of mitochondrial membrane potential in the LO2 cells (A) and BRL-3A cells (B) determined by fluorescence microscopy. (d) Representative pictures of mitochondrial reactive oxygen species (mROS) level in the LO2 cells (A–D) and BRL-3A cells (E–H) determined by fluorescence microscopy. (e) Triglyceride (TG) content in the LO2 cells. (f) Immunoblotting and statistical analysis of p-AMP-dependent protein kinase (p-AMPK) and AMPK in the LO2 cells pretreated with Nrf2 inhibitor ML385. Data are presented as the means ± SEM. ^∗∗^*P* < 0.01, comparison between the indicated groups.

In order to further verify that 4-OI reduces FFA-induced ROS accumulation by activating the Nrf2 signaling pathway, subsequently, hepatocytes were preincubated with 10 *μ*M Nrf2 inhibitor ML385. As shown in Figures 6(a) and 6(b), 4-OI induced the upregulation of Nrf2 protein level and the downregulation of Keap1 protein level markedly reversed in the FFA-stimulated LO2 cells and BRL-3A cells by pretreatment with ML385. Moreover, we found that the increase of nuclear Nrf2 protein level induced by 4-OI treatment obviously dispelled in the FFA-stimulated LO2 pretreatment with ML385 (Figure 6(a)). Mitochondrial membrane potential (MMP) and mitochondrial ROS (mROS) levels were measured by JC-1 and DCFH-DA fluorescent probe, the representative images showed that 4-OI treatment significantly attenuated the increase of mROS production and the decrease of MMP level in the FFA-stimulated LO2 cells and BRL-3A cells, and these effects was blocked in cells pretreatment with ML385 (Figures 6(c) and 6(d)). Importantly, we also found that ML385 inhibited the increase of phosphorylation AMPK protein level induced by 4-OI treatment in the FFA-stimulated LO2 cells (Figure 6(f)). Meanwhile, the reduced action on TG content induced by 4-OI treatment was also completely reversed in the FFA-stimulated LO2 cells pretreated with ML385 (Figure 6(e)). These results demonstrated that 4-OI inhibits the overproduction of mROS through the activated Nrf2, which subsequently activates AMPK signal pathway to inhibit the lipid accumulation in the FFA-stimulated hepatocytes.

## 4. Discussion

**Figure 7:**
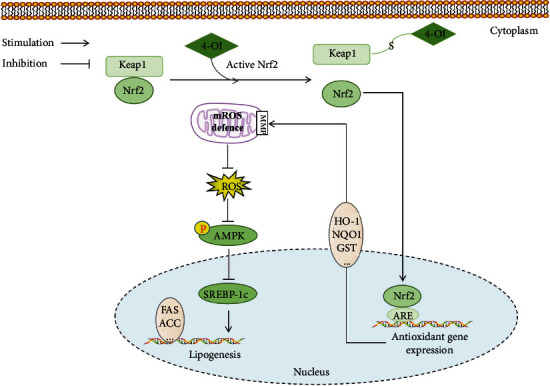
Schematic illustration of the mechanism of 4-OI alleviating lipid metabolism disorders by activating the Nrf2-AMPK signaling pathway. 4-Octyl itaconate (4-OI) enhances the antioxidant function through activating the nuclear factor E2-related factor 2 (Nrf2), which leads to the increase of mitochondrial membrane potential (MMP) and inhibition of the reactive oxygen species (ROS) overproduction in free fat acid- (FFA-) stimulated hepatocytes. Meanwhile, the downregulation of ROS level induces an increase of AMP-dependent protein kinase (AMPK) phosphorylation level, which alleviates the lipid deposition in free fat acid- (FFA-) stimulated hepatocytes. In conclusion, 4-OI relieves the oxidative stress and lipid metabolism disorder by activating the Nrf2-AMPK signaling pathway in FFA-stimulated hepatocytes.

Numerous studies have shown that excessive intake of free fatty acids can lead to abnormal lipid deposition in hepatocytes and then lead to oxidative stress and inflammatory response, which may exacerbate liver steatosis [28–31]. Therefore, alleviating abnormal lipid deposition and oxidative stress becomes an effective strategy for the prevention of NAFLD. Previous study had found that treatment with 4-OI can protect against oxidative stress and inflammatory response *in vivo* and *in vitro* [17]. However, whether 4-OI can alleviate hepatic steatosis and oxidative stress and its mechanism is still unknown. In this study, we demonstrated that 4-OI alleviates FFA-induced lipid metabolism disorder in hepatocytes by activating AMPK signaling pathway; importantly, the activation of AMPK pathway was achieved via Nrf2-mediated inhibition of mROS production in the FFA-stimulated hepatocytes (Figure 7). These results provided a new insight for 4-OI used as a lipid-lowering regulator to protect lipid metabolism disorders and related diseases.

Under normal physiological conditions, lipid synthesis and lipolysis are in homeostasis [32]; and once this dynamic is broken due to increased synthesis and/or weakened decomposition of lipids, it may lead to excessive lipid accumulation [33, 34]. The lipid droplet accumulation and TG content in the cells are important parameters for assessing lipid deposition [35]. In the present study, we found that 4-OI treatment significantly reduced the lipid droplet accumulation and TG content in the FFA-stimulated LO2 cells and BRL-3A cells, which implied that 4-OI can alleviate lipid accumulation in the FFA-stimulated hepatocytes. As a metabolic regulatory factor, activated AMPK can reduce lipid deposition through inhibiting lipid synthesis-related factors expression and enhancing lipolysis-related factors expression [36]. In this study, 4-OI significantly reversed the reduction of AMPK and ACC1 phosphorylation levels and the increase of SREBP-1 protein levels in hepatocytes induced by FFA-stimulated. As a key downstream target factor of AMPK, ACC1 is a rate-limiting enzyme in the fatty acid synthesis pathway [37], and its activity is inhibited by its phosphorylation [38]. In order to further clarify whether AMPK plays a crucial role in 4-OI mediated lipid lowering in hepatocytes, the AMPK inhibitor compound C was used to block the AMPK phosphorylation. We observed that the activation of AMPK signaling pathway induced by 4-OI treatment was observably reversed in the FFA-stimulated hepatocytes pretreated with compound C. Meanwhile, compound C pretreatment also blocked the effects of 4-OI on reducing the mRNA or protein levels of SREBP-1c and FAS and promoting the *PPARα* and *CPT-1* mRNA levels in the FFA-stimulated hepatocytes. SREBP is an important nuclear transcription factor that regulates the genes expression related to lipid synthesis [39, 40]; as one of the subtypes, SREBP-1c is mainly responsible for activating the expression of lipid synthesis genes [41]. In addition, acetyl-CoA can be catalyzed by ACC1 to generate malonic acid monoacyl CoA, which finally is catalyzed by FAS to produce fatty acids [42]. Taken together, these data indicated that 4-OI alleviates the lipid deposition by activation of AMPK signaling pathway in the FFA-stimulated hepatocytes.

Mitochondria are the main organelle that generates endogenous ROS [43]. In mitochondria, electrons flow to complex IV in an orderly manner along the electron transport chain and react with oxygen-containing substances at several electron transfer points to produce ROS [44]. Excessive lipid accumulation can abate the mitochondrial respiratory chain enzymes activity, change mitochondrial membrane permeability, and induce excessive production of ROS [45, 46]. The homeostasis between the production and clearance of ROS is the basis for maintaining normal cell functions [47]. In present study, we found that 4-OI reduced the ROS overproduction in the FFA-stimulated hepatocytes. As a signal molecule regulating biochemical pathways, the excessive production of ROS will destroy the body's oxidation defense system [48]. Our also found that FFA induced an increase of MDA content, while 4-OI treatment significantly reduced MDA content and increased the total antioxidant capacity in the FFA-stimulated hepatocytes. MDA level is a marker to measure the effect of lipid peroxidation, and the decrease of MDA content is also an indicator to improve antioxidant performance [49]. Moreover, we found that FFA induced the inhibition of the activities of SOD, CAT, and POD alleviated in hepatocytes treated with 4-OI. These results implied that 4-OI improved the antioxidant capacity and attenuated the ROS accumulation in the FFA-stimulated hepatocytes.

The damage is caused by oxidative stress involved in all aspects of metabolism [50]. As a classic antioxidant pathway, the Keap1-Nrf2 system is the major regulatory pathway for cytoprotective gene expression against oxidative stresses [51, 52]. In this study, 4-OI treatment obviously increased the expression of Nrf2 protein and its downstream genes. These results are consistent with the previous reported by Mills et al. [17]. Moreover, our results found that the increase of Nrf2 protein level and decrease of Keap1 protein level induced by 4-OI treatment were reversed in cells pretreated with Nrf2 inhibitor ML385. It has been reported that 4-OI acts as an activator of Nrf2 to alkylate cysteine residues on Keap1 and break the connection between Keap1 and Nrf2, which finally results the accumulation of Nrf2 in nuclear [17, 20]. In addition, our results showed that 4-OI ameliorated the decrease in mitochondrial membrane potential caused by overproduction of ROS in the FFA-stimulated hepatocytes. Thus, we speculate that 4-OI can improve the mitochondrial function through reducing ROS accumulation in the FFA-stimulated hepatocytes. Zorov et al. reported that high level of ROS can disrupt the normal function of mitochondria and produce more ROS [53, 54]. Meanwhile, mitochondrial membrane potential is the result of mitochondrial oxidative phosphorylation [54] and the basis of mitochondrial ATP production [55]. These results implied that 4-OI relieves oxidative damage by activating the Nrf2 signaling pathway that maintains the mitochondrial membrane potential and reduces the excessive production of mROS in the FFA-stimulated hepatocytes.

It is remarkable that excessive production of ROS induced by mitochondrial dysfunction can also lead to lipid metabolism disorders [56]. Therefore, we used ROS scavenger NAC to detect the AMPK signaling pathway in the hepatocytes. We observed that NAC alone treatment significantly alleviated the reduction of AMPK and ACC1 phosphorylation in the FFA-induced hepatocytes. Meanwhile, we used Nrf2 inhibitor ML385 to investigate the effect of Nrf2 on the activation of AMPK pathway. Interestingly, we found that ML385 significantly reversed the increase of AMPK phosphorylation level induced by 4-OI treatment in the FFA-stimulated hepatocytes. Meanwhile, the decreasing effect of 4-OI on TG content was also completely reversed in the FFA-stimulated hepatocytes pretreatment with ML385. These results demonstrated that 4-OI treatment activated AMPK pathway via the Nrf2-mediated inhibition on mROS production, which subsequently inhibited the lipid accumulation in the FFA-stimulated hepatocytes. Although we have provided *in vivo* evidence to demonstrate the role of 4-OI in regulating oxidative stress and lipid metabolism disorders, the specific mechanism deserves further study in combination with animal models. In addition, itaconate alkylates cysteine residues in proteins, including GAPDH, LDHA, and SDH except of Keap1, and these modifications inhibit glycolysis and TCA cycle, which are important for the supply of cytosolic acetyl-CoA for fatty acid synthesis. Whether the regulation of lipid metabolism by 4-OI is also affected by glucose metabolism requires further research to explore this process in more detail.

## 5. Conclusion

Our data demonstrated that 4-OI enhances the antioxidant function through activating the Nrf2, which leads to the increase of MMP and inhibition of the ROS overproduction in the FFA-stimulated hepatocytes. Meanwhile, the downregulation of ROS level induces an increase of AMPK phosphorylation level, which alleviates the lipid deposition in the FFA-stimulated hepatocytes. In conclusion, our findings indicated that 4-OI relieves the oxidative stress and lipid metabolism disorder by activating the Nrf2-AMPK signaling pathway in the FFA-stimulated hepatocytes. These data provide a theoretical basis for 4-OI as a nutritional supplement in the prevention and treatment of metabolism disorders and related diseases.

## Figures and Tables

**Table 1:** Sequences of target genes and GAPDH primers.

Target gene	Primers sequence
*SREBP-1c*	F 5′-GTGTATGTGCCCTCTGTGCT-3′
	R 5′-CTCCCAAAGTGCCTGACAGA-3′
*FAS*	F 5′-AGGCCTCATAGACCTGCTGA-3′
	R 5′-GGGAGATGAGGGGAGTTCCT-3′
*CPT-1*	F 5′-ATCAATCGGACTCTGGAAACGG-3′
	R 5′-TCAGGGAGTAGCGCATGGT-3′
*PPARα*	F 5′-ACGATTCGACTCAAGCTGGT-3′
	R 5′-GTTGTGTGACATCCCGACAG-3′
*HO-1*	F 5′-GGCCAGCAACAAAGTGCAAGA-3′
	R 5′-TAAGGACCCATCGGAGAAGCG-3′
*NQO1*	F 5′-CGCAGACCTTGTGATATTCCAGT-3′
	R 5′-GGTCCTTTGTCATACATGGCAGC-3′
*GAPDH*	F 5′-CCTGCACCACCAACTGCTTAG-3′
	F 5′-CAGTCTTCTGGGTGGCAGTGA-3′

## Data Availability

The [DATA TYPE] data used to support the findings of this study are available from the corresponding author upon request.
